# Osteogenic potential of silver nanoparticles in critical sized mandibular bone defects: an experimental study in white albino rats

**DOI:** 10.1007/s10266-024-01049-2

**Published:** 2025-01-10

**Authors:** Gehad Mohamed Sabry, Nessma Sultan, Mazen Tharwat Abouelkhier, Essam Farouk Soussa

**Affiliations:** 1 Oral Biology Department, Faculty of Dentistry, Mansoura University, Mansoura, Egypt; 2 Lecturer Oral Biology and Dental Morphology, Faculty of Dentistry, Mansoura National University, Gamasa, Egypt

**Keywords:** Silver nanoparticles, Hydroxyapatite nanoparticles, Critical size defect, Animal model, Osteoregeneration

## Abstract

Natural bone is a self-regenerating nanocomposite made of proteins and minerals. Such self-regenerative capacity can be negatively affected by certain diseases involving the bone or its surrounding tissues. Our study assesses the ability of bone grafting material to regenerate bone in animals who have artificially created critical-sized defects. Nanohydroxyapatite (HANPs) and silver nanoparticles (AgNPs) were synthesized and underwent characterization by transmission electron microscopy. The cytotoxic effect of the nanomaterials was evaluated by MTT assay using bone marrow mesenchymal stem cells (BMMSCs). Five mm critical size defects in white albino rats were utilized to assess the material’s biocompatibility, and regenerative capacity. Histological and immunohistochemical analyses using collagen-I and tumor necrotic factor-alpha were also performed. Clinically, the tested materials did not cause any pathological changes. MTT results suggested that both materials showed high biocompatibility. Gel form of AgNPs achieved bone regenerative potential and anti-inflammatory effect being significantly higher than what was seen in HANPs after 21 days post-surgically. The utilization of AgNPs to improve anti-inflammatory and osteoregenerative activities was the primary research outcome of this study. AgNPs have the potential to be useful biomaterial in accelerating bone healing process.

## Introduction

Critical size defect (CSD) is a proven way to assess tissue-engineered structures in vivo. When considerable bone loss is seen following trauma, cancer or infection surgery, or both, the CSD model simulates clinical settings. Using CSD as an experimental model, the effectiveness of recently developed biomaterials in promoting bone formation has been evaluated prior to therapeutic application. CSD is defined as the smallest intraosseous wound in a specific bone that does not heal on its own throughout the animal’s lifetime [1–3]. The healing phase of CSD is protracted and time-consuming because of inadequate blood supply to the fracture site and insufficient minerals (calcium and phosphorus) to increase the strength and hardness of the newly formed bone [4]. Moreover, fibroblasts migrate to the wound site in such inadequacies faster than osteoblasts because fibrous connective tissue shows a quicker regeneration than bone tissue [5, 6].

The mechanism behind the cellular activity leading to wound healing at the CSD site was actually the release of tissue factors such as osteogenin or bone morphogenetic protein from the wound’s edge [7]. As a result, cells in the defect become differentiated into osteoblasts and chondroblasts, which generate the extracellular matrix and mineralize it, forming bony islands. The creation of new bone is supported by these islands, which function as a scaffold. However, the low prevalence of differentiation can be partly attributed to the scarcity of tissue components in the middle of the CSD. The gradual replacement of chondrocytes and osteoblasts by fibrous connective tissue occurs when they are unable to mineralize the matrix [3].

Nanoparticles have extraordinary mechanical properties because of their surface, volumetric, and quantum effects [8]. Because of their distinct structural properties, drug transport, and biocompatibility, nanoparticles have been thoroughly evaluated for their bone regeneration potential [9, 10]. Since hydroxyapatite nanoparticles (HANPs) have outstanding biocompatibility, bioactivity, and osteoconductivity, they are widely employed as inorganic biomaterials enhancing bone regeneration [8].

Silver nanoparticles (AgNPs) have demonstrated a strong antibacterial activity and the capacity to prevent the formation of biofilms in a number of investigations conducted in recent years as a small amounts of silver ions are continuously released by silver metal, inhibiting the growth of germs on the metal surface [11, 12]. Despite being one of the noble metals and thus only mildly reactive, silver’s physicochemical characteristics may not match those of the bulk metal in nanoparticle form. An enhanced surface to volume ratio at the nanoscale causes an increase in silver release. As such, it is imperative to properly explore the mechanism of nanotoxicology and to carefully assess the risks that silver may pose at the nanoscale. There is currently insufficient knowledge regarding AgNPs’ potential effects on bone tissue regeneration. Thus, in this study, we evaluated the effect of AgNPs on the bioactivity of bone-forming cells and mesenchymal stem cells (MSCs) in comparison to the widely used inorganic HA nanomaterials in alveolar bone augmentation in rat’s CSD in the mandible.

## Material and methods

### Experimental design and sample size calculation

The current study was performed on a total of 54 male, pathogen-free, albino rats weighting 180–200 g. All experimental procedures were performed according to the protocol that obtained its approval from the Ethical Committee of Faculty of Dentistry, Mansoura University, Egypt with number A11030821. In a light-controlled room, rats were kept at 22 °C constant temperature with a 12:12-h light–dark cycle and a relative humidity of 65–70%. Animals received commercial diet and water. All rats were monitored for at least 1 week preoperatively. They were randomly allocated into 3 groups (*n* = 18); group I (control group) where mandibular bone defect was left empty, group II where mandibular bone defect was grafted with HANPs, and group III where mandibular bone defect was grafted with AgNPs in a gel form. The group size was based on power analysis based on our published data (histologic analysis) with a 5% significance level and a power of 95%. Based on previous publications [13, 14], 6 animals/group/time point were normally sufficient.

### Materials formulation

Rat bone marrow mesenchymal stem cell line (BMMSCs) was purchased from Cyagen OriCell-RAWMX-01001. The cell line was already characterized according to the manufacture instructions.

#### Synthesis of HANPs

HANPs were prepared by chemical precipitation using calcium nitrate tetrahydrate (Samchun Chemicals, Seoul, Korea) and diammonium hydrogen phosphate (Samchun Chemicals, Seoul, Korea) according to the method described by *Mondal, 2016* [15]. The synthesized HANPs underwent dryness at 70 ^◦^C and calcination at 600 ^◦^C for 1 h in the air atmosphere.

#### Synthesis of AgNPs hydrogel

AgNPs were synthesized by photo irradiation method [16]. AgNPs-loaded hydrogels were fabricated. Briefly, 3% w/v concentrated aqueous solution of gelatin mixed at 400 rpm for 1 h at 40 °C. Following cooling to the room temperature, filtration of the solution was performed using 0.22 µm syringe filter, then it was combined with 1% w/v concentrated silver nitrate solution with stirring at 400 rpm for 10 min. The mixture was exposed to 354 nm long UV waves for 1h (JENAWAY6305, UK spectrophotometer).

### Characterization of nanoparticles

#### Transmission electron microscope (TEM)

The size and morphologic properties of HANPs and AgNPs were characterized by TEM (TEM, JEOL 1200EX, Japan) operating at 200 kV. The NPs were dropped on the carbon-coated 400-mesh copper TEM grid and underwent complete dryness prior to TEM examination.

#### MTT assay for cell viability

The cell viability was determined with a Vybrant^®^ MTT cell proliferation assay kit. In brief, BMMSCs were seeded in a 96-well cell culture plate. After 24–48 h, different nanomaterial concentrations (5, 10, 25, 50, 100 µM) were applied to the culture medium of BMMSCs and then underwent incubation in the CO2 incubator for 72 h. A negative control of medium without cells was used as a blank. The medium was discarded and replaced with a culture medium that was freshly prepared without phenol red. MTT reagent was added to the cells which underwent incubation at 37 °C for 2–4 h. Dimethyl sulfoxide was added to each well to dissolve in soluble forming formazan. Finally, mixing of all samples was done and absorbance was determined at 570 nm (BioTek ELx 800) (HIGHLAND PARK, US).

### Surgical procedure

**Fig. 1:**
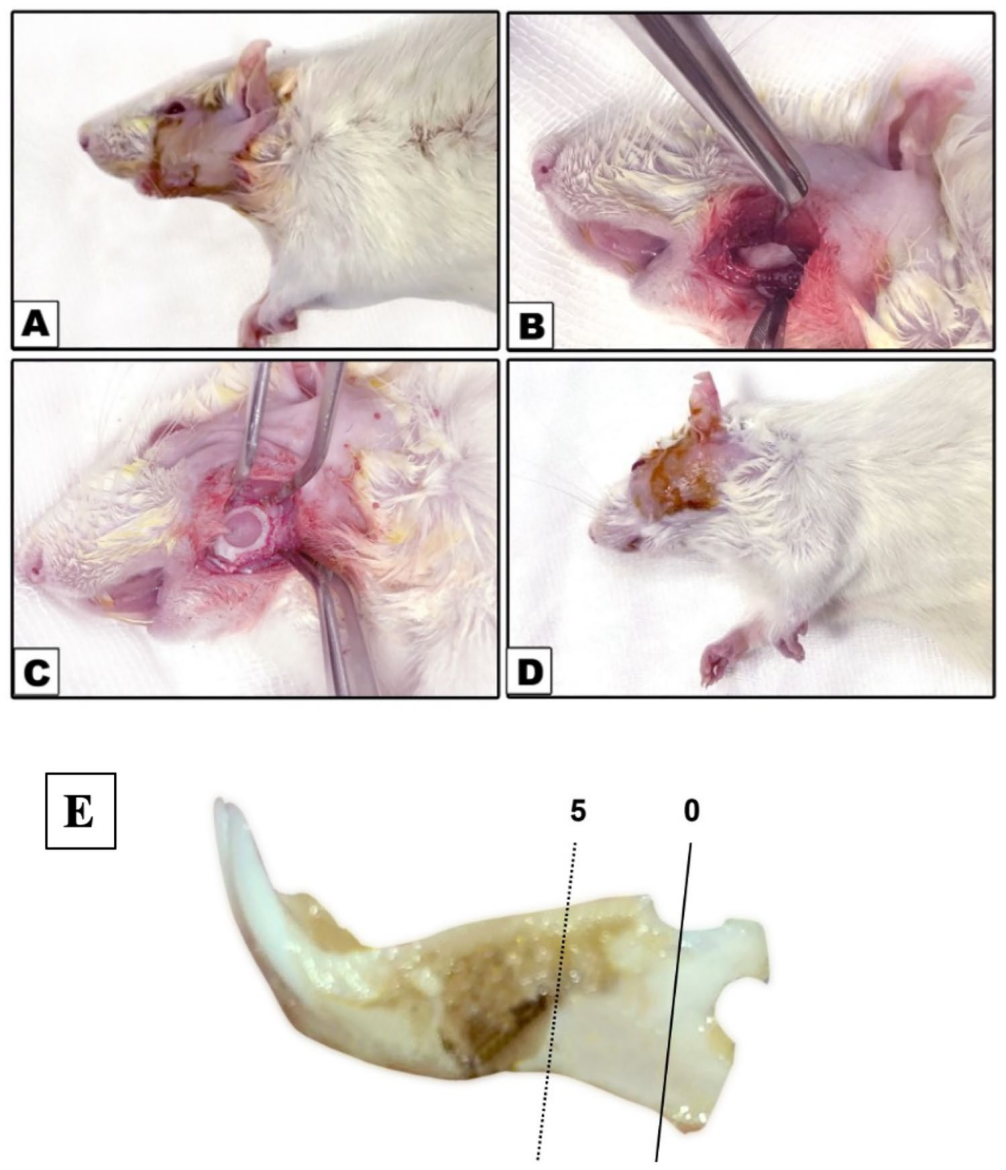
Photographs showing step-by step surgical procedure for creating the CSD in the mandible. E showing the exact location of the CSD in rat’s mandible

Prior to each surgery, animal body weight was measured. The surgery was carried out under sterile and aseptic conditions. First, the rat was anaesthetized using a mixture of ketamine (Virvac, Nice, France) (40–90 mg/kg) and xylazine (Rompum, Bayer, Leverkusen, Germany) (5–10 mg/kg) [17]. Then the skin on the right side of the mandible was shaved and then disinfected using a sterile cotton pellet wet with povidone-iodine 10% (Betadine^®^) (Fig. 1).

A submandibular incision was made through the skin, subcutaneous tissue, and masseter muscle paralleling the inferior border of the mandible. The buccal surface of the mandible was exposed with an elevator, and 5 × 5 mm and 1 mm depth critical defects were created in the body of the mandible in the right side in each animal (Fig. 1E). Defects were created with a trephine bur (with inner diameter 5 mm and outer diameter 6 mm (3i, Palm Beach Gardens, FL, USA) in a slow-speed dental drill (NSK, latch bur, Tokyo, Japan). To prevent over-heating, cooling with saline was dropped onto the contact point between the bur and bone. A periodontal probe was used to measure the bone defect to ensure standardized size [18]. After bone defect formation, it was filled with the tested nano materials (15 mg of powder HANPs, while AgNPs was used as a gel form and 25 µl was used to fill the defect). Soft tissue above the defect was closed with 4–0 absorbable Vicryl sutures (Ethicon, Johonson & Johonson, Somerville, NJ, USA), a sterile cotton pellet wet with Betadine^®^ was used to disinfect the operation site.

### Post-operative care

After recovery from anesthesia, rats were placed in clean, dry recovery cages, each animal was kept on 150 mg/kg of body weight amoxicillin IM twice daily (E-MOX^®^ 250 mg, Egyptian International Pharmaceutical Industries, Ramadan, Egypt) and analgesics (Voltaren 75 mg/3 mL Novartis, Giza, Egypt) for 3 days [19].

### Euthanasia

Six rats from each group were euthanized after 3, 14, 21 days post-surgically using overdose of halothane. Then their mandibles were harvested for histological assessment.

### Specimen processing and histological assessment

Once specimen have been harvested and cleaned carefully from any adherent soft tissues, they were fixed in 10% neutral-buffered formalin for 48 h. Specimens were then washed under running tap water, then they were decalcified using 10% EDTA solution. After confirming adequate decalcification, segments of interest were trimmed, then processed and embedded in wax blocks for histological assessment [20].

#### Hematoxylin and eosin stain (H&E)

For routine assessment of the bone structure, hematoxylin dye that stains nucleic acid in the cell nucleus a purplish blue and eosin an acidic dye that stains proteins in the cytoplasm and extracellular matrix pink were used.

#### Immunohistochemistry (IHC)

Using a conventional streptavidin–biotin immunoperoxidase approach, collagen-I antibody (COL-I; Cat# NB600-408**,** Novus Biologicals) and tumor necrotic factor-alpha (TNF-alpha; Cat# Ab307164, Abcam) were used for the immunohistochemical examination. Staining procedures were performed as sections were deparaffinized in xylene and hydrated with gradient ethanol ethyl alcohol. The enzyme recovery was performed in EDTA over 3 min in a pressure cooker. Blocking of endogenous peroxidase with H_2_O_2_ (10 min) was followed by the blocking of non-specific binding (20 min) in BSA/PBS. Incubation of slides was done overnight at pre-diluted concentrations (1:100) of the specific antibodies according to the manufacturer instructions.

### Image analysis

After excluding the rats that showed complications, a total of 50 tissue slides were prepared from the control and experimental groups; 17 for control group, 16 for HANPs-, and 17 for AgNPs-treated groups. Six slides were prepared for control and experimental groups at 3 days, five tissue slides were prepared at 7 days and while at 21 days, five tissue slides prepared for HANPs and six tissue slides for either control or AgNPs-treated groups. Four randomly selected areas in the middle of each CSD were captured at 100 × magnification. Then twenty to twenty-four images for each group per time point were used for analysis. The ratio of newly formed bone to the total defect area in the images was calculated. Image analysis was carried out using the Intel^®^ Core I7^®^ based computer and VideoTest Morphology^®^ software (Version 5.0, Russia). It features a specific built-in routine for area and percentage area measurements. Images were stained with COL-I and TNF-alpha where the positive reaction appeared as brown color.

### Statistical analysis

Each defect was used as a statistical unit and one defect was prepared in each rat; thus, six statistical units were assigned for each group at three different time points (*n* = 18). Data were expressed as mean ± SEM. Data were analyzed by SPSS with one-way ANOVA and Tukey’s post hoc test. Significance levels were set at * *p* < 0.05, ** *p* < 0.01, *** *p* < 0.001, and **** *p* < 0.0001. The threshold of significance was set at 5% level.

## Results

### Animals and clinical observations

Out of the 54 rats included in this study, 4 rats were excluded from the analysis: one from control group, one from AgNPs group, and two from HANPs group. The excluded rats from AgNPs- and HANPs-treated groups showed post-surgical complications. While one rat of the control group developed an abscess on the post-operative day 7 and it was killed humanely once the abscess was discovered.

### Characterization of nanoparticles materials by TEM

**Fig. 2:**
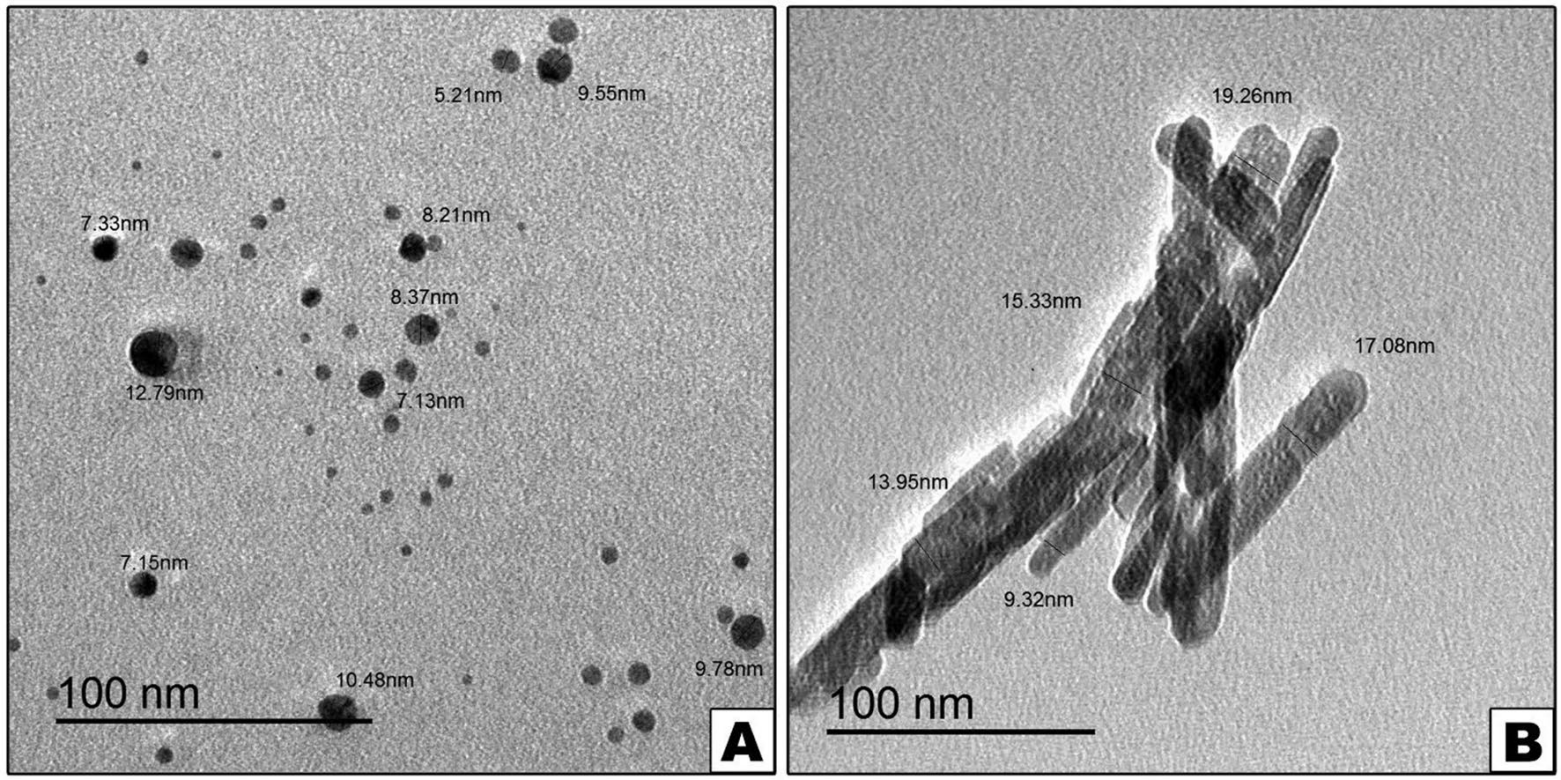
Showing TEM micrographs for the nanomaterials used in the study, where **A** showing AgNPs, and **B** showing HANPs

TEM micrographs of the AgNPs demonstrated distinct, uniformly spherical shapes that were well-separated from each other. The mean particle size of AgNPs ranged between 2 and 12 nm (Fig. 2A). TEM images of HANPs showed that the particles were having a rod-like structure. It was found that the size of HANPs was 10–32 nm (Fig. 2B). The mean diameter of around 500 particles on TEM micrographs was measured to determine grain size distribution.

### Cytotoxicity of nanomaterials by MTT assay

**Fig. 3:**
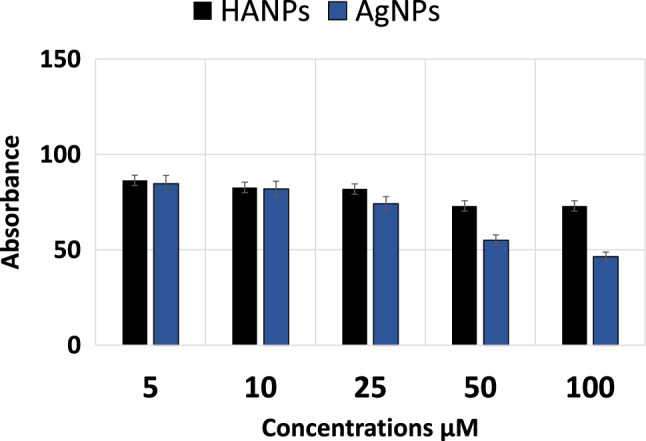
MTT analysis of viable BMMSCs number cultured with the tested nanomaterial at different concentrations for 72 h. Data are presented as mean ± SD; *n* = 3

MTT assay was utilized to test the cytotoxicity of the tested nanomaterials. Different concentrations of the nanoparticle’s suspension were used to treat BMMSCs. Cell viability was determined at 72-h post-treatment and the results are demonstrated in Fig. 3. It has been noticed that cell viability decreases as the concentration of tested materials increases. HANPs showed non-significant difference in the cell viability among different concentrations while, AgNPs showed significant reduction in the viable cell number with increased concentrations at 50 and 100 µM. Hence, the concentration of 10 µM has been found to maintain high cell viability in the tested nanomaterials and this concentration was used in all other experiments.

### Histological results

A total of 50 CSDs were made available to be analyzed microscopically after excluding the rats experienced complications. The distinct contrast between the old and the newly formed woven bones, characterized by abundant osteocytes, enabled a clear identification along the border of the defect. CSDs were sliced into cross-sections and H&E, COL-I, and TNF-alpha stains were applied to enable a detailed analysis of the regeneration pattern and to confirm a successful bone regeneration.

#### H&E results

##### After 3 days

**Fig. 4:**
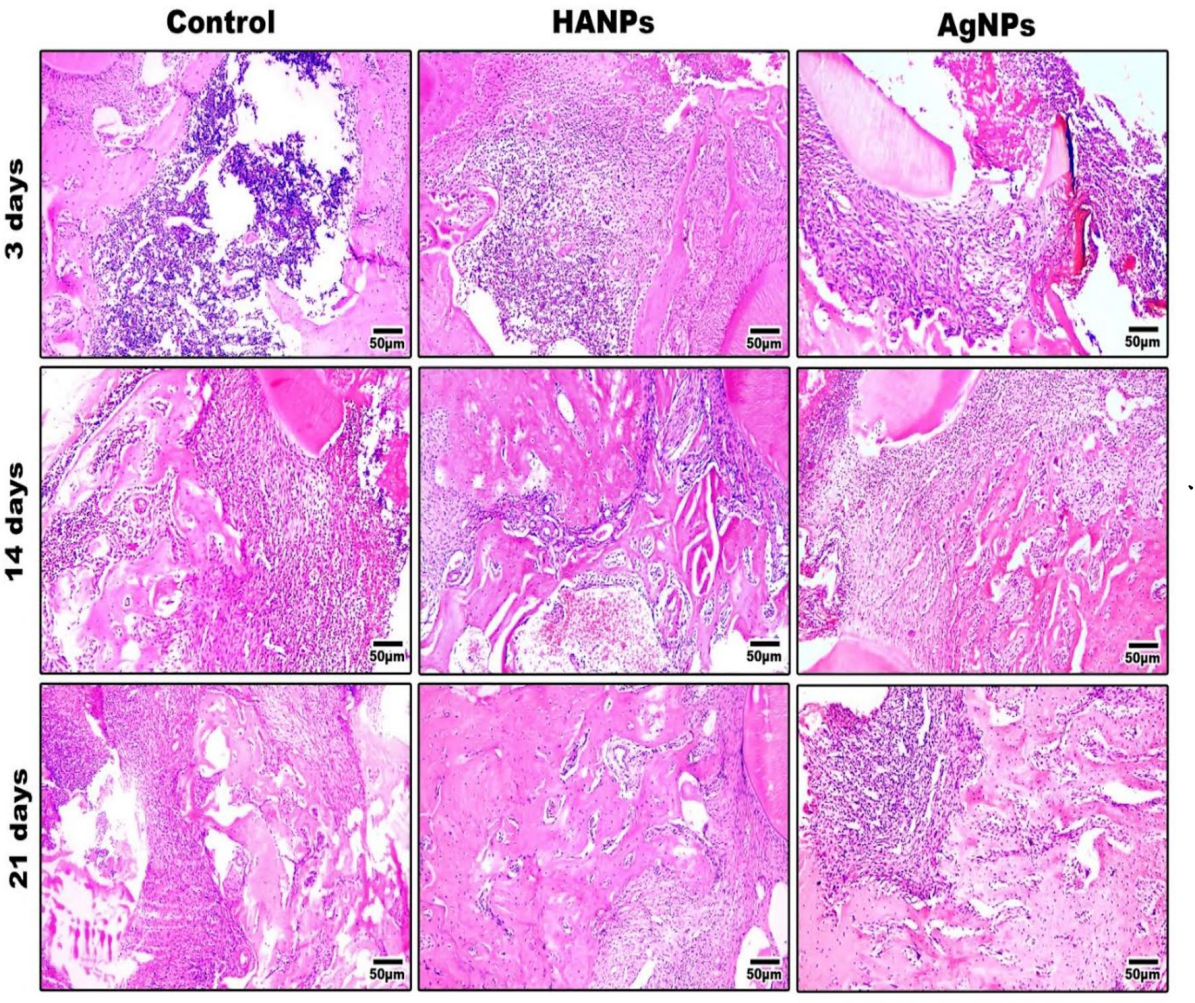
Representative histological images of (H&E) staining for the tested groups after 3, 14 and 21 days. Fibrous connective tissues were seen to be mostly filled in the defect areas in the control group over the tested time points. The group treated with HANPs showed a newly formed bone bridging the defect area at 14 days post surgically which increased obviously after 21 days. AgNPs- treated group showed considerable new bone trabeculae formation after 14 days which increased after 21 days with abundant number of osteocytes. Scale bar: 50 μη

In control animals, an irregular, thin layer of fibrous connective tissue was observed filling the CSD, without any new-formed bone. In HANPs-treated animals, osteoblasts were seen lining the bone trabeculae between predominantly fibrous connective tissues. Partially aligned connective tissue was observed in the defect, without significant new bone formation at its margin. The AgNPs-treated animals showed woven bone formation at the CSD edge with osteocytes within the bone trabeculae, confirming an active bone formation. (Fig. 4).

##### After 14 days

In control animals, CSDs were filled with fine fibrous connective tissues with thin bone trabeculae formed. The HANPs-treated group showed uniformly dense and well-aligned connective tissues in the CSD with a new bone formation were observed. The AgNPs group showed woven bone formation bridging the margin of the CSD. (Fig. 4).

##### After 21 days

In control animals, the CSD still filled with granulation tissue with a very thin newly formed bone trabecula. HANPs- and AgNPs-treated groups revealed significant amounts of new bone that covered the center of the defect. There was a great amount of newly formed bone in both groups with substantial number of osteocytes. The formed woven bone progressively bridged the defect, signifying a gradual and promising bone maturation.

#### Immunohistochemical staining results

##### COL-I stain results

**Fig. 5:**
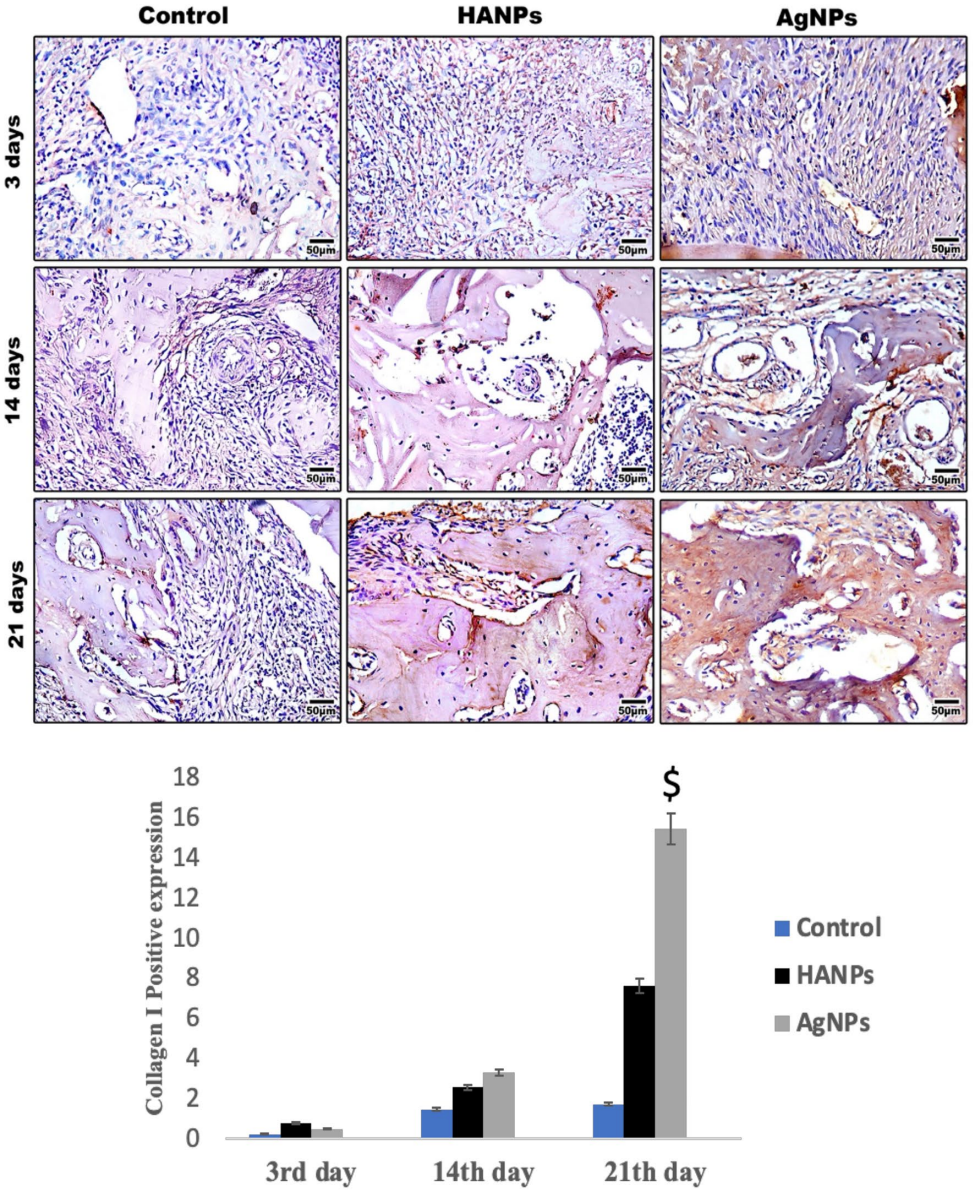
Representative immunohistochemical images of COL-I antibody are shown at 3-, 14- and 21-days post-surgically. Histological sections through the mid-point of the defects were prepared from decalcified specimens. Fibrous connective tissues are mostly filled in the defect areas in the control group after 3 days while over 14- and 21-days post-surgically, thin bone trabeculae started to be formed. The groups treated with HANPs and AgNPs materials showing fibrous tissue at 3 days of treatment while an observed newly formed lamellar bone with strong COL-I expression appeared after 21 days of treatment. Scale bar. 50 µm. Bar charts revealed the histomorphometric analysis of COL-I positive expressions at different time points. Data are expressed as the mean ± SEM. Where $ means significant in relation to all other groups

For quantification of the degree of bone regeneration, the new bone in the defect was evaluated with COL-I staining to calculate the percentage of positive expression (brown color). In control animals, the expression was 0.245 ± 0.03, 1.47 ± 0.44 and 1.73 ± 0.44 at 3, 14, and 21 days, respectively. For the HANPs group, the expression was 0.789 ± 0.35, 2.57 ± 0.38, and 7.64 ± 0.50 at 3, 14, and 21 days, respectively. In the AgNPs group, the expression was 0.492 ± 0.15 after 3 days and 3.30 ± 0.048 after 14 days, while reached 15.49 ± 0.79 after 21 days (Fig. 5). These results suggest variable degrees of bone regeneration between the different studied groups, with AgNPs demonstrating the most promising outcome.

COL-I expression showed marked increase in the AgNPs group from 14 to 21 days post-surgically. Noteworthy that after 14 days, no significant difference in COL-I expression was found between the AgNPs group and HANPs group, whereas after 21 days, the positive expression of COL-I was significantly higher among the AgNPs-treated animals compared to that among the HANPs-treated animals. This suggests that AgNPs can substantially affect bone regeneration and advancement, predominantly evident in the extended period. At day 3, no significant difference in COL-I expression was found between control and AgNPs-treated groups whereas control group showed significance in comparison to HANPs after 3 days post-surgically. The significance between control and AgNPs-treated group became more obvious after 14 and 21 days (p < 0.01). Noteworthy, no significant difference in COL-I expression was found in the control group between 14 and 21 days; however, in the treated groups, the COL-I expression significantly increased over time.

##### TNF-alpha stain results

**Fig. 6:**
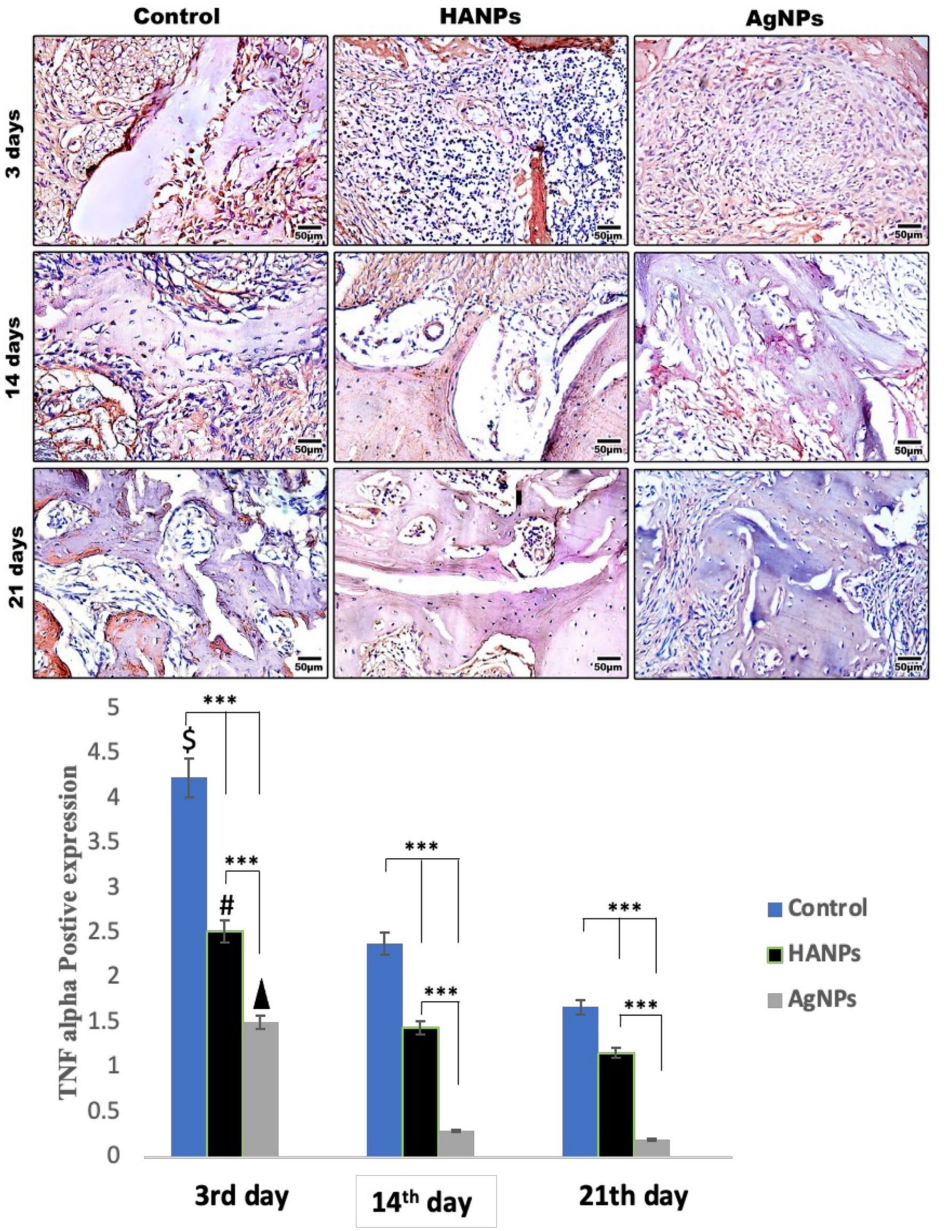
Representative immunohistochemical images of TNF-alpha antibody are shown at 3, 14 and 21 days after treatment. Histological sections through the mid-point of the defects were prepared from decalcified specimens. The expression of the inflammatory immune marker was mostly filled in the defect areas in the control group. The groups treated with HANPs and AgNPs materials showing marked reduction in TNF-alpha expression at 14 and 21 days being significantly reduced in AgNPs treated groups revealing its anti-inflammatory effect. Scale bar: 50 µm. Bar charts revealed the histomorphometric analysis of TNF-alpha positive expressions at different time points. Data are represented as the mean ± SEM. Where $ means significant in relation to control after 14 and 21 days, ▲ means significant in relation to HANPs after 14 and 21 days, means significant in relation to AgNPs after 14 and 21 days. ***P < 0.001

To quantify the inflammatory degrees, TNF-alpha immune marker was used. Calculation of the percentage of positive expression (brown color) showed that the expression was 4.23 ± 0.39, 2.38 ± 0.55 and 1.67 ± 0.37 at 3, 14, and 21 days, respectively, in control animals. For the HANPs group, the expression was 2.52 ± 0.38, 1.44 ± 0.13, and 1.16 ± 0.13 at 3, 14, and 21 days, respectively. In the AgNPs group, the expression was 1.50 ± 0.47 after 3 days and 0.29 ± 0.10 after 14 days, while reached 0.19 ± 0.05 after 21 days. In HANPs group, no significant difference in TNF-alpha expression was found between 14 and 21 days; a similar trend was noticed in AgNPs group. There was no statistical significance between 14 and 21 days which suggests the need to extend the time point over 21 days. However, a significant reduction in TNF-alpha expression was noted after 3rd day in all the studied groups being significantly reduced in AgNPs-treated group (Fig. 6).

## Discussion

Nanomaterials have a key role in bone tissue engineering research [8]. While bone can undergo self-regeneration and remodeling in many cases such as infection, severe trauma, tumor or cyst resection, external convention is still necessary [1, 2]. In this study, HANPs and AgNPs were chosen to investigate their effects on bone healing of a surgically induced 5 mm mandibular bone defect. Rats were used as the study model because their bone structure is similar to human bone, and they are inexpensive, easy to house, and readily available.

The characteristic features of nanoparticles, including size, shape, size distribution, should be assessed before evaluating toxicity or biocompatibility [21]. TEM is a structural and characterization technology with great spatial resolution that gives precise information about the size and shape of particles [22, 23]. The AgNPs have a spherical shape and a diameter of 2–12 nm. The reported spherical shape of the AgNPs highly agrees with the study by Mock et al.  [24].

A cytotoxic effect of the tested nanomaterials was measured using BMMSCs in vitro. BMMSCs were exposed to different concentrations of nanomaterials and it was noticed that cell viability reduced as the concentration of materials was increased. The cytotoxic effect of nanomaterials was proven to be based on the size the NPs, the smaller the particle size, the weaker the cytotoxic effect and higher antibacterial activity [25]. This indicates the importance of customizing the size of NPs to optimize toxicity and antibacterial activities. From this perspective, the obtained NPs meet this criterion, with a diameter of AgNPs 2–12 nm and HANPs 10–32 nm.

In terms of the histological analysis, the CSDs in control animals were filled with granulation tissue; at 14 days after surgery, a small amount of delicate osteoid tissue was discovered around the defect; and at 21 days, thin, newly formed bone trabeculae began to show up without the defect being completely filled.

HA is a naturally occurring apatite mineral that is present in hard tissues, including vertebrates’ teeth and bones. It is well-known for having strong osteoconductivity and osteoinductivity. Since HA occurs in nanoscaled tissue in nature, n-HA is synthesized and used to increase the bioactivity of biomaterials [26, 27]. The cycle of bone resorption by osteoclasts and bone synthesis by osteoblasts resumes when the area of a bone defect is filled by HA, resulting in the development of new autologous bone [28]. In this study, using a nanometer-sized granules increases the activity of osteoblasts, leading to an enhanced proliferation, adhesion, and differentiation of bone cells. Bone defect filled with granulation tissue at 3 days, then part of granulation tissue replaced with woven bone of thin trabeculation after that thicker bone trabeculation was found without complete filling of bone defect with bone at 21 days [29].

On the other hand, the use of AgNPs for bone regeneration as an osteoinductive material is limited; however, it was extensively used as anti-inflammatory or antibacterial agent. In AgNPs-treated group, the results showed greater amount of woven bone tissue and osteocytes bridging the defect area at 14 and 21 days post-surgically. Our results agreed with previously published studies [30, 31] which confirmed the osteogenic role of AgNPs illustrating that the enhanced osteogenesis was probably related to the induction of callus formation, and the closure of fracture gap by 21 days after implantation through chemotaxis of BMMSCs and fibroblasts to reach the fracture site, initiation of proliferation and differentiation of local BMMSCs adjacent to the fracture site.

In bone, COL-I is predominantly produced by osteoblasts, which also control the production of hydroxyapatite from deposited calcium and phosphate salts. In our study, COL-I expression was evaluated in the tested groups and it was found that HANPs results revealed significant upregulation of COL-I expression over the tested time points [31]. A similar trend was noticed in AgNPs-treated groups where COL-I expression increased gradually being significantly increased after 21 days in comparison to control and HANPs groups which revealed a potent osteoinductive potential of AgNPs in comparison to the widely used HANPs.

Bone fractures are injuries associated with inflammation. Inflammatory signals promote the local increase in macrophages, which release cytokines that are important for the healing process. In the present study, immunohistochemical staining of TNFα which assessed inflammatory response of nanomaterials revealed that there was a strong inflammatory response in control group in the first 3 days post-surgically [32]. A reduction in the inflammatory response was found in HANPs group, these results reflect anti-inflammatory effect of HANPs [33, 34]. AgNPs-treated group demonstrated the least expression level of inflammatory response in comparison to the studied groups, this may be as a result of the inherent anti-inflammatory property of AgNPs [35, 36] suggesting that modulation of local inflammatory reactions can promote bone healing.

## Conclusion

In conclusion, our study displays the anti-inflammatory effect of AgNPs together with its impact in improving the osteogenesis process in the CSDs of rat’s mandible. However, the osteoinductive potential of AgNPs needs to further be analyzed over long time period to predict the possible health risks.

## Data Availability

The data obtained over the course of the investigation can be accessed after contacting the corresponding author.
